# Early versus late transition to oral antibiotics in Enterobacterales bacteremia

**DOI:** 10.1017/ash.2026.10886

**Published:** 2026-09-29

**Authors:** Musa Yahia, Cassandra Falk, Raymond Yost, Abigail Geyer

**Affiliations:** Pharmacy, Henry Ford Wyandotte Hospital, Wyandotte, USA

## Abstract

**Objective:**

Evaluate outcomes of early versus late intravenous (IV)-to-oral antibiotic transition in patients with uncomplicated Enterobacterales bacteremia.

**Design:**

Multicenter, retrospective observational cohort study.

**Setting:**

Healthcare system with one tertiary care academic medical center and four community teaching hospitals.

**Patients:**

Adults with uncomplicated Enterobacterales bacteremia, clinical stability within 72 hours of antibiotic initiation, definitive source control, ability to take oral medication, and final susceptibility reports were included. The following were excluded: never received oral antibiotic; inadequate treatment duration; transitioned back to IV therapy; inappropriate antimicrobial therapy based on dosing or susceptibilities; transferred from outside institution; immunocompromised; pregnant.

**Methods:**

Early transition was considered IV-to-oral antibiotic transition by day 4 of therapy. The primary outcome was treatment failure within 30 days of antibiotic completion, a composite end point encompassing infection recurrence, escalation of antimicrobial therapy, infection-related re-admission, and death. Secondary outcomes included hospital length of stay and total antibiotic duration.

**Results:**

225 patients were included; 133 (59.1%) transitioned to oral antibiotics early and 92 (40.9%) transitioned late. There were no significant differences in the primary composite outcome of treatment failure for early transition versus late transition (6.0% vs 6.5%, *P* = .877). Early transition was associated with shorter median hospital length of stay (5 vs 7 d, *P* < .001) and total antibiotic duration (9 vs 12 d, *P* < .001).

**Conclusion:**

Early IV-to-oral antibiotic transition in patients with uncomplicated Enterobacterales bacteremia and clinical stability within 72 hours was effective with potential to reduce length of stay and total antibiotic duration.

## Introduction

Enterobacterales bacteremia, a serious and common bloodstream infection caused by gram-negative bacteria including *Escherichia coli*, *Klebsiella pneumoniae* or *oxytoca*, and *Proteus mirabilis*, constitutes a significant proportion of bloodstream infections.^1^ Historically, patients with uncomplicated gram-negative bacteremia received 14 days of parenteral antimicrobial therapy which has been associated with several complications including catheter-related infections, extended hospital stays, and increased healthcare costs.^2–4^ However, previous evidence has demonstrated that clinically stable patients can be safely transitioned from intravenous (IV) to oral antibiotic therapy, providing an opportunity to reduce the burden associated with catheter-related an opportunity to reduce the burden associated with prolonged IV treatment. ^5–8^ As evidence supporting oral step-down therapy has grown, recent studies have increasingly focused on determining how early this transition can safely occur. Engers and colleagues found that most patients with gram-negative bloodstream infections achieved clinical stability within 5 days of antibiotics, yet fewer than half transitioned to oral therapy within 7 days, suggesting an opportunity for earlier conversion.^9^ Alzaidi and colleagues evaluated oral transition within 4 days of effective IV therapy and found similar clinical effectiveness between trimethoprim-sulfamethoxazole and fluoroquinolones, while Mateu and colleagues reported no significant differences in mortality or relapse among selected patients transitioned to oral therapy by day 3 or earlier compared with those who received later transition or continued IV therapy.^10,11^ These findings are significant for antimicrobial stewardship and continued advocation for timely IV-to-oral conversions to potentially reduce therapy costs, experience fewer IV-related complications, and improve patient quality of life.

Despite increasing evidence supporting IV-to-oral conversion, the optimal timing of the transition remains uncertain, particularly whether earlier transition provides comparable clinical outcomes to later transition. This knowledge gap leaves clinicians without clear guidance or consensus on the optimal transition approach. Therefore, the purpose of this study is to evaluate the outcomes of early versus late oral antibiotic transition in patients with uncomplicated Enterobacterales bacteremia. We hypothesized that clinically stable patients transitioned earlier to oral antibiotics would have no difference in treatment failure compared to those transitioned later to oral antibiotics for the treatment of Enterobacterales bacteremia.

## Methods

### Study design and setting

This was a multicenter, retrospective cohort study of adult patients treated for uncomplicated Enterobacterales bacteremia at a large health system between September 1, 2021, and September 1, 2024. The health system included one tertiary care academic medical center, 3 community teaching hospitals, and 1 community hospital. The study protocol was approved by the Institutional Review Board with a waiver of informed consent.

### Inclusion and exclusion criteria

Eligible participants were adults 18 years or older with uncomplicated Enterobacterales bacteremia (Figure 1) who had definitive source control, if applicable, and achieved clinical stability within 72 hours of appropriate IV antimicrobial initiation (Figure 2). Definition of clinical stability was developed utilizing The Consensus Statements for Best Practices for the Management of Uncomplicated Gram-Negative Bloodstream Infections criteria.^13^ Therefore, we defined clinical stability as achievement of at least 3 of 4 criteria: Temperature ≤38°C, HR <90/min, RR <20/min, hemodynamic stability (SBP > 90 mmHg). Patients additionally had to be eligible for transition to oral antibiotics (Figure 2), have a final antibiotic susceptibility report available for the infecting organism, and be discharged alive without hospice or palliative care.

**Figure 1.**
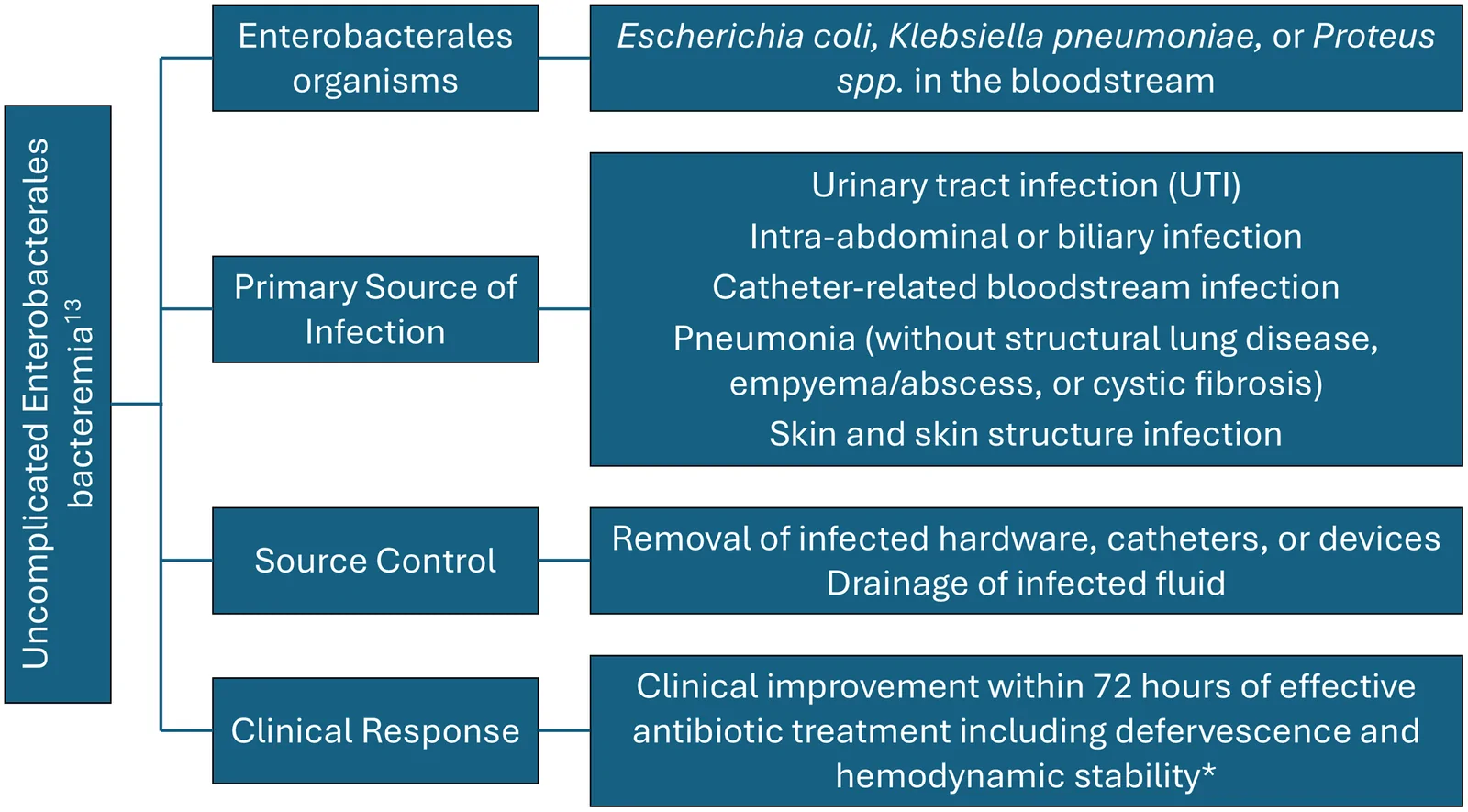
Definition of uncomplicated Enterobacterales bacteremia. *Clinical stability defined as achievement of at least 3 of 4 criteria: Temperature ≤38°C, HR <90/min, RR <20/min, hemodynamic stability (SBP > 90 mmHg).

**Figure 2.**
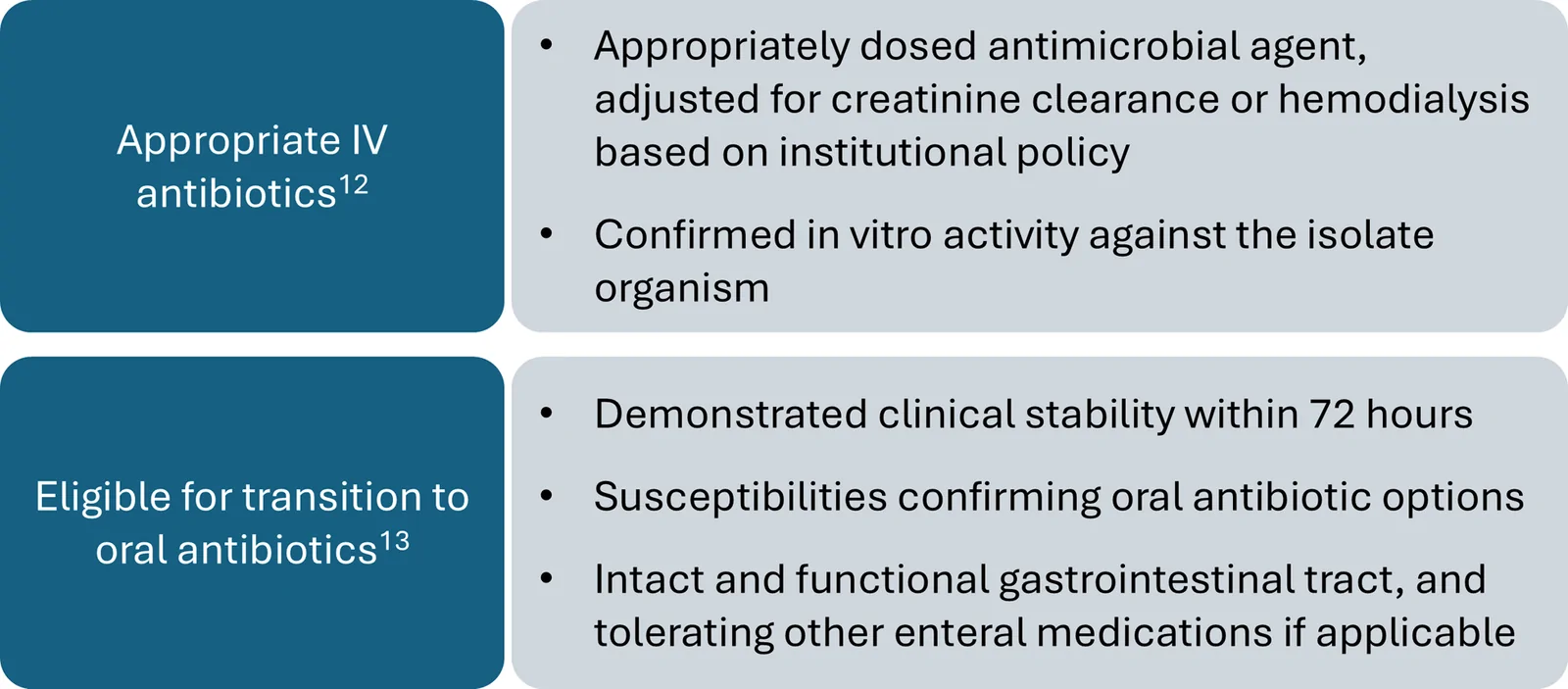
Definitions of eligibility criteria.

Patients were then excluded for the following reasons: never transitioned to oral therapy during inpatient admission, had polymicrobial infection with non-Enterobacterales organisms, received less than 7 days of appropriate antimicrobial therapy, transitioned back to IV antibiotics, received inappropriate antimicrobial therapy based on dosing chosen or susceptibilities, transferred from an institution outside of the health system, or were pregnant or incarcerated. Patients were also excluded if they were immunocompromised as defined by having received any solid organ transplant, expected prolonged neutropenia (ANC <500 cells/mL), CD4 cell count <200 cells/mL, or currently on chronic corticosteroids and/or immunomodulator therapy.^13^ Exclusions are outlined in Figure 3.

**Figure 3.**
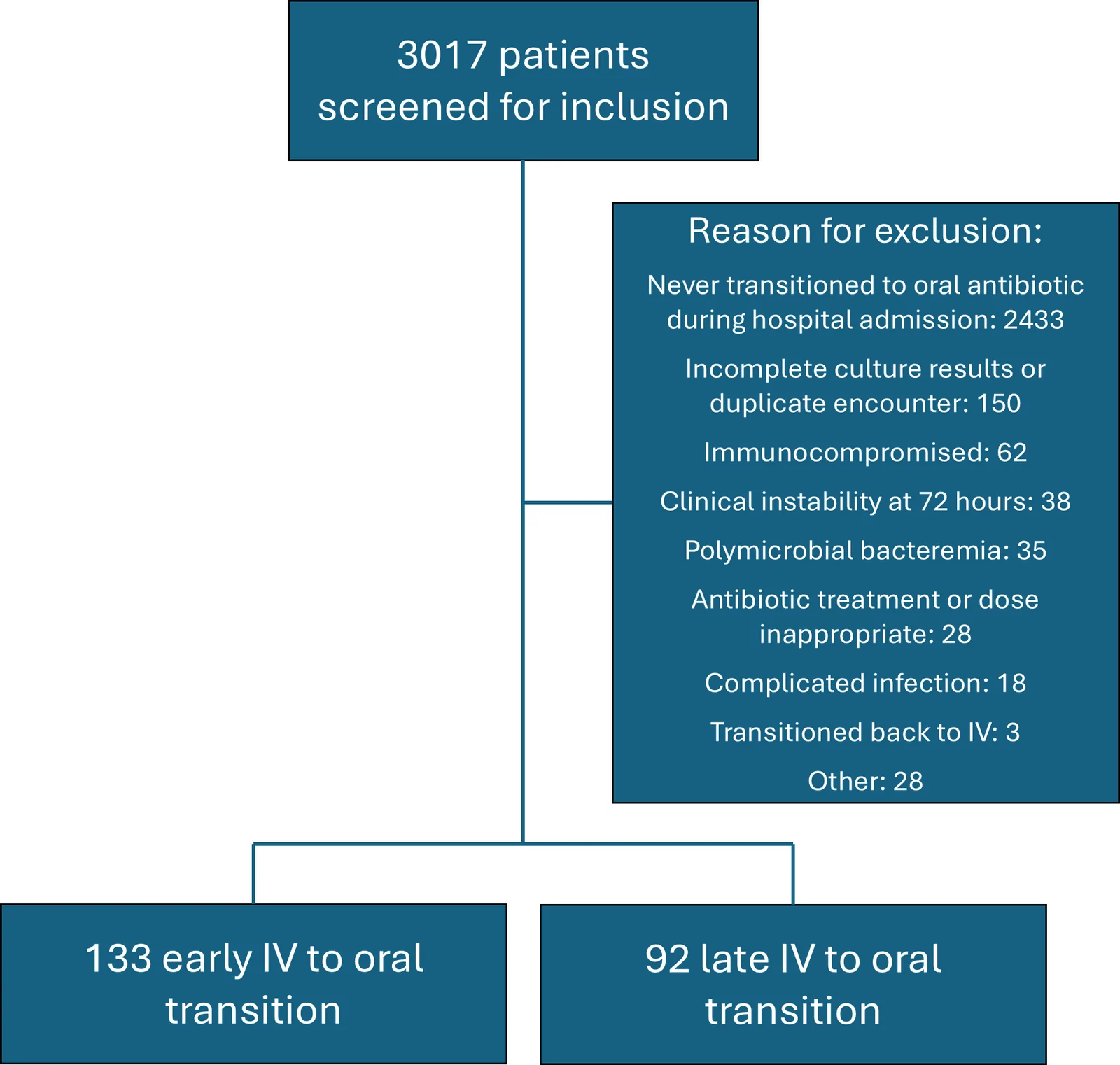
Screening and enrollment.

Patients were separated into two groups based on the timing of their transition from IV-to-oral antibiotics: an early transition group, defined as patients transitioned on day 3 or 4 of antibiotic therapy, and a late transition group, defined as patients transitioned on day 5 or later of antibiotic therapy.

### Data collection

Patients were identified from blood cultures positive with any of the three predefined isolates within the study time frame and digitally abstracted from the electronic health record (EHR) system, Epic. Data pertaining to patient demographics, clinical presentation, and outcomes were manually collected from the EHR. Laboratory results, microbiological findings, antibiotic treatment regimens, *International Classifications of Disease* (ICD) codes used to calculate the Charlson Comorbidity Index (CCI) were also extracted digitally from Epic and confirmed through manual patient chart review. Standardized data collection forms were utilized to ensure consistency and completeness of data abstraction, and a separate process was implemented to ensure the de-identification of all patient information to maintain confidentiality. All data was collected by the primary investigator, and sub-investigators audited 10% of charts throughout data collection to verify the accuracy of information digitally extracted and manually obtained from patients’ EHR.

### Study outcomes

The primary outcome of the study was treatment failure within 30 days of completing the prescribed course of oral antibiotics. This was a composite end point defined as the occurrence of one or more of the following: the need for additional antimicrobial therapy, infection-related readmission to the hospital, microbiological relapse, or all-cause mortality. The need for additional antibiotic therapy was defined as a broadening in coverage of the patient’s current antimicrobial regimen or the initiation of a new antibiotic. Microbiological relapse was defined as recurrent bacteremia with the same organism. Secondary outcomes included the incidence of each component of the composite primary outcome, total hospital length of stay measured in days from admission to discharge, and the total duration of antibiotic therapy. Duration of antibiotic therapy was measured as the sum of IV and oral antibiotic days. Antimicrobial days were counted using the total number of calendar days in which a patient receives any amount of a specific antimicrobial agent as outlined by NHSN.^14^

### Statistical analysis

Using 80% power and an alpha of 0.05, it was estimated that 125 patients would be needed in each group to detect a 10% difference in treatment failure. A greater difference in treatment failure was assumed compared to what Rieger et al observed due to differences in timing of IV-to-oral antibiotic transition and treatment failure definition between studies.^6^

Statistical analysis was performed using IBM SPSS Statistics 28.0.1. Categorical variables were analyzed using Pearson *χ* ^2^ or Fisher’s Exact tests, as appropriate. Continuous variables were analyzed using the Mann–Whitney test for non-parametric data. Statistical significance was set as a *P* value <.05. To address potential confounding variables, clinically relevant risk factors for treatment failure and risk factors that had a bivariate analysis *P* value <.2 were considered for multivariate logistic regression.

## Results

### Participants

A total of 3,017 patients 18 years or older with Enterobacterales bacteremia were screened for inclusion (Figure 3). After all exclusions, the final cohort consisted of 225 patients. Of these, 133 (59.1%) were categorized into the early IV-to-oral transition group and 92 (40.9%) in the late IV-to-oral transition group.

### Study population

Baseline characteristics are shown in Table 1. Patients in the late IV-to-oral transition group presented with a higher burden of comorbidities, evidenced by a significantly higher median CCI (5 vs 6, *P* = .027). Furthermore, individuals in the late transition group were more likely to have an infectious diseases (ID) consult (57.1% vs 70.7%, *P* = .039) and to have been admitted to the intensive care unit (ICU) (21.1% vs 45.7%, *P* < .001). All other characteristics were similar between the groups. Patients were primarily Caucasian, over the age of 65, with genitourinary tract infections and *Escherichia coli* as the predominant infecting organism.

**Table 1.** Baseline characteristics Table 1 long description.

Characteristic	Early IV-to-oral transition (n = 133)	Late IV-to-oral transition (n = 92)	*P*
Age (yr), median [IQR]	74 [64–85]	76 [68–83]	.459
Gender at birth, n. (%)			
Female	77 (57.9)	57 (62)	.542
Race, n (%)			
White	106 (79.7)	74 (80.4)	.892
Black	21 (15.8)	14 (15.2)	.907
BMI ≥ 35 kg/m^2^, n. (%)	32 (24.1)	23 (25)	.872
Charlson comorbidity index, median [IQR]	5 [3–7]	6 [4–8]	.027
Comorbidities, n. (%)			
Chronic obstructive pulmonary disease	25 (18.8)	28 (30.4)	.043
Diabetes mellitus	49 (36.8)	40 (43.5)	.317
Renal insufficiency	17 (12.8)	21 (22.8)	.048
Antimicrobial allergies, n. (%)			
None	102 (76.7)	60 (65.2)	.064
TMP/SMX	4 (3)	8 (8.7)	
Cephalosporin	2 (1.5)	0 (0)	
Penicillin	15 (11.3)	11 (12)	
FQ	1 (0.8)	5 (5.4)	
Multiple antimicrobial allergies	9 (6.7)	8 (8.7)	
Infectious diseases consult, n. (%)	76 (57.1)	65 (70.7)	.039
ICU admission, n. (%)	28 (21.1)	42 (45.7)	<.001
Causative pathogen, n. (%)			
* Escherichia coli*	105 (78.9)	62 (67.4)	.250
* Klebsiella spp.*	16 (12)	18 (19.6)	
* Proteus spp.*	10 (7.5)	9 (9.8)	
Multiple Enterobacterales	2 (1.5)	3 (3.3)	
Source of infection, n. (%)			
Genitourinary	111 (83.5)	67 (72.8)	.211
Intra-abdominal	15 (11.3)	20 (21.7)	
Respiratory	3 (2.3)	2 (2.2)	
Skin/soft tissue	3 (2.3)	1 (1.1)	
Other	1 (0.8)	2 (2.2)	

BMI, body mass index TMP-SMX, trimethoprim-sulfamethoxazole FQ, fluoroquinolone.

Vital signs at baseline and after 72 hours of IV antibiotics are shown in Table 2. At baseline, the late transition group had a higher proportion of patients with a respiratory rate greater than 20 breaths per minute (BPM), higher median white blood cell (WBC) count, and higher median lactate. At 72 hours, the late transition group continued to have a higher proportion of patients with a respiratory rate greater than 20 BPM and higher median WBC count.

**Table 2.** Vitals and labs Table 2 long description.

	Early IV-to- Oral Transition (n = 133)	Late IV-to-Oral Transition (n = 92)	*P*
**Baseline**			
Temperature >38°C or <36°C, n. (%)	69 (51.9)	42 (45.7)	.358
HR greater than 90 BPM, n. (%)	81 (60.9)	62 (67.4)	.320
RR greater than 20 BPM, n. (%)	64 (48.1)	58 (63)	.027
WBC in cells/µL, median [IQR]	12.4 [9.3–17.4]	14.8 [10.8–20.5]	.011
Lactate in mg/dL, median [IQR]	1.9 [1.3–3.1]^*^	2.4 [1.7–4.1]**^+^**	.001
**72 h after antimicrobial therapy initiation**			
Temperature >38°C or <36°C, n. (%)	4 (3)	2 (2.2)	1.000
HR greater than 90 BPM, n. (%)	24 (18)	23 (25)	.207
RR greater than 20 BPM, n. (%)	18 (13.5)	31 (33.7)	<.001
WBC in cells/µL, median [IQR]	8.9 [6.3–10.5]	9.6 [7.6–13.4]	.002
Lactate in mg/dL, median [IQR]	No recorded values for any patients at 72 h		**–**

BPM, beats or breaths per minute RR, respiratory rate HR, heart rate WBC, white blood count.

* 123 patients.

+ 87 patients.

### Intravenous antibiotic regimens

Table 3 outlines all antibiotic treatment regimens. Ceftriaxone was the most frequently used IV antibiotic in both groups. Additionally, within our sample, 118 of 225 (52.4%) patients were treated with multiple IV antibiotics. These patients experienced a change in therapy from one or multiple empiric broad-spectrum agents to a definitive agent. 110 of these 118 (93.2%) patients transitioned definitively to ceftriaxone, while 8 (6.8%) patients remained on broad-spectrum therapy. Of the patients transitioned to ceftriaxone, 83 (75.5%) transitioned from piperacillin-tazobactam or cefepime, 14 (12.7%) from carbapenem, 6 (5.5%) from aztreonam, and 7 (6.4%) from ciprofloxacin.

**Table 3.** Treatment characteristics Table 3 long description.

	Early IV-to-oral transition (n = 133)	Late IV-to-oral transition (n = 92)	*P*
Intravenous antibiotics prescribed, n (%)			
Multiple antibiotics	55 (41.4)	63 (68.5)	.007
Ceftriaxone	69 (51.9)	28 (30.4)	
Piperacillin-tazobactam	4 (3)	1 (1.1)	
Aztreonam	2 (1.5)	0 (0)	
Ertapenem	1 (0.8)	0 (0)	
Ciprofloxacin	1 (0.8)	0 (0)	
Ampicillin-sulbactam	1 (0.8)	0 (0)	
Oral antibiotics prescribed, n. (%)			
Cephalosporin	35 (26.3)	43 (46*.7*)	.010
TMP/SMX	44 (33.1)	17 (18.5)	
Fluoroquinolone	32 (24.1)	17 (18.5)	
Penicillin	15 (11.3)	13 (14.1)	
Multiple antibiotics	7 (5.3)	2 (2.2)	

TMP/SMX, trimethoprim/sulfamethoxazole.

### Oral antibiotic regimens

Table 3 outlines all antibiotic treatment regimens. Trimethoprim/sulfamethoxazole (TMP/SMX) was the most frequently prescribed oral agent in the early transition group, while cephalosporins and fluoroquinolones were more frequently used in the late transition group. Additionally, within our sample, 9 of 225 (4%) patients were treated with multiple oral antibiotics. 7 of 9 (77.8%) patients received TMP/SMX followed by either oral fluoroquinolone, cephalosporin, or penicillin.

### Outcomes

All primary and secondary outcomes are outlined in Table 4. There was not a statistically significant difference in treatment failure between the early and late transition groups (6.0% vs 6.5%, *P* = .877). No single component of the primary composite outcome contributed to treatment failure either above the rest. For secondary outcomes, the early transition group had a shorter median hospital length of stay (5 vs 7 d, *P* < .001) and a shorter median total antibiotic duration (9 vs 12 d, *P* < .001).

**Table 4.** Primary and secondary outcomes Table 4 long description.

	Early IV-to-oral transition (n = 133)	Late IV-to-oral transition (n = 92)	*P*
**Primary outcome**			
Treatment failure within 30 d, n (%)	8 (6)	6 (6.5)	.877
Escalation of/additional antibiotic therapy	5 (3.8)	3 (3.3)	1.000
Infection-related readmission	3 (2.3)	2 (2.2)	1.000
Microbiologic relapse	2 (1.5)	1 (1.1)	1.000
All-cause mortality	3 (2.3)	3 (3.3)	.691
**Secondary outcomes**			
Hospital length of stay, median [IQR]	5 [4–6.5]	7 [6–10]	<.001
Duration of treatment, median [IQR]			
Oral antibiotic duration	6 [4–8]	6 [3–9]	.598
Intravenous antibiotic duration	3 [3–4]	5 [5–6]	<.001
Total antibiotic duration	9 [8–11]	12 [9–14]	<.001

### Bivariate risk factor analysis

Results of the bivariate analysis assessing potential risk factors for treatment failure can be found in Table S1 within the supplementary index. Multivariate logistic regression was not indicated based on these results.

## Discussion

Despite a higher baseline severity of illness in the late transition group based upon Charlson comorbidity scores and increased ICU admission, patients who were clinically stable at 72 hours experienced no difference in treatment failure if transitioned to oral antibiotics early versus late. These results align with previous studies suggesting that early oral transition can be safe and effective in select patients with gram-negative bacteremia. Tamma and colleagues included patients with monomicrobial Enterobacterales bacteremia caused by any infectious source. Their findings demonstrated that oral antibiotic therapy, when initiated by day 5 in clinically stable patients, did not affect 30-day mortality in comparison to IV-only treatment.^8^ Tingsgård and colleagues contributed further to this area of research. Patients with monomicrobial Enterobacterales bacteremia who were clinically stable and transitioned to oral antibiotics within 4 days of their initial blood culture had similar 90-day mortality rates compared to patients receiving prolonged IV therapy which was defined as at least 5 days of IV antibiotics.^5^ Additionally, Rieger and colleagues compared outcomes for patients transitioned to oral antibiotic therapy versus patients on IV-only therapy. Patients in this study had Enterobacterales bacteremia originating only from urinary tract infections, and patients transitioned to oral antibiotics received a median of 4 days of IV antibiotics. No significant difference in 30-day treatment failure rates was observed, defined as escalation to IV from oral antibiotic, change in treatment due to worsening clinical status, or readmission for the same infection and pathogen.^6^ More recently, Mateu and colleagues evaluated very early oral transition at day 3 or earlier compared to later oral transition or all IV antibiotic course. This study found very early transition was safe and effective for stable patients with Enterobacterales bacteremia, however, longer time to clinical stability impeded early oral transition.^11^ Our study differs because instead of comparing IV-to-oral transition to IV-only therapy, we expand upon current literature by assessing early oral transition versus later oral transition. None of the patients in our study received IV-only therapy. This distinction is critical, as there is a lack of consensus and data guiding the optimal timing for IV-to-oral conversion in this population.

Additionally, secondary outcomes demonstrated potential advantages for patients transitioned to oral antibiotics early, including a shorter hospital length of stay and a reduced total duration of antibiotic therapy. This could be driven by prescriber comfortability to discharge patients or utilize shorter durations of therapy if a patient has already been switched to oral therapy during their hospitalization. However, we cannot state this with certainty because prescriber comfortability is not a variable that can be directly collected upon retrospective chart review. Previous literature describes a shorter hospital length of stay by 2–3 days with IV-to-oral antibiotic transition compared to prolonged IV therapy or IV-only therapy.^6–8^ Our study aligns with these findings and additionally highlights shorter antibiotic treatment courses when patients were transitioned early to oral therapy (median 9 vs 12 d). For the patients who received more than 7 days of antibiotics for uncomplicated Enterobacterales bacteremia, excess duration seemed to be driven by prolonged discharge prescription durations as our median hospital length of stay was 5 days (early IV-to-oral group) and 7 days (late IV-to-oral group).

As for our bivariate risk factor analysis assessing potential risk factors for treatment failure, there were no results compelling to proceed with multivariate logistic regression. None of the variables had a *P* value < .2 for model inclusion, and other variables of interest were either protective or had no effect. We anticipated the presence of an ID consult to be a major confounding variable for our primary outcome of treatment failure. We felt this was a clinically relevant variable to assess as there were a higher proportion of patients in the late transition group who received ID consults. However, our bivariate risk factor analysis suggested no impact on treatment failure (unadjusted OR 1.08; 95% CI 0.35–3.33). Another confounding variable of interest that did not have a *P* value < .2 was elderly age, but bivariate risk factor analysis suggested no impact on treatment failure (unadjusted OR 1.01; 95% CI 0.33–3.13). Lastly, the later transition group had a higher proportion of patients with ICU admission which was hypothesized to affect our primary outcome, but our risk factor analysis suggests that ICU admission would reduce treatment failure (unadjusted OR 0.35; 95% CI 0.08–1.61). Given the results of bivariate risk factor analysis, multivariate logistic regression was not performed.

## Limitations

There are limitations of this study that need to be considered. First, as a retrospective study, we relied on accurate documentation in the EHR to obtain data. We had multiple people verify information digitally extracted and manually obtained from patients’ EHR. Second, our sample size was small causing our study to be underpowered and potentially reduced external validity. Based upon previous literature by Reiger et al, we needed 125 patients in each group to detect a 10% difference in treatment failure, which we did not achieve.^6^ However, our multicenter approach encompasses real-world practice from five diverse sites within a health system, which may enhance its applicability to varied practice settings. Additionally, although we attempted to establish defined criteria for “clinical stability” and “uncomplicated” bacteremia, some degree of subjective interpretation by the reviewer could have influenced patient inclusion or exclusion based on these criteria. Furthermore, our study population predominantly involved uncomplicated urinary tract infections. Although this is consistent with previous research, it may restrict the direct applicability of our findings to more complex or severe infections. However, this approach minimized confounding from higher-risk patients or more severe infection sources. Lastly, we did not explore factors associated with early transition to oral antibiotics like previous works.^9,11^ Engers and colleagues described clinical characteristics associated with prolonged IV therapy to include the following: higher CCI, on immunosuppression, hypotension, or fevers by day 5, ICU admission, and kidney replacement therapy.^9^ Additional factors impeding early oral transition were identified by Mateu and colleagues including extended spectrum beta-lactamase enzyme producing isolates and lack of source control.^11^ Future studies with larger sample sizes would be warranted to better identify areas for targeted education and antimicrobial stewardship.

Overall, in clinically stable patients with uncomplicated Enterobacterales bacteremia, early transition on day 3 or 4 of antimicrobial therapy from IV-to-oral antibiotics was not associated with increased rates of treatment failure compared to transitioning later on day 5 or beyond of therapy. However, early transition could potentially reduce hospital length of stay and overall antibiotic duration. These findings support the safety and clinical benefits of early oral step-down, reinforcing it as a key strategy in antimicrobial stewardship. Further prospective research is warranted to validate these findings and aid in the establishment of clear guidelines for optimal patient selection and timing of IV-to-oral antibiotic transitions.

## Supporting information

10.1017/ash.2026.10886.sm001 Yahia et al. supplementary material Yahia et al. supplementary material

## Supplementary material

The supplementary material for this article can be found at https://doi.org/10.1017/ash.2026.10886.

## Table 1. Long description

The table compares baseline characteristics of patients in early and late IV-to-oral transition groups. It has 18 rows and 5 columns. The columns are labeled ‘Characteristic’, ‘Early IV-to-oral transition (n = 133)’, ‘Late IV-to-oral transition (n = 92)’, and ‘P’. The characteristics include age, gender at birth, race, BMI, Charlson comorbidity index, comorbidities, antimicrobial allergies, infectious diseases consult, ICU admission, causative pathogen, and source of infection. Each row provides specific data for these characteristics. Notable trends include higher median Charlson comorbidity index, more infectious diseases consults, and higher ICU admission rates in the late IV-to-oral transition group.

Navigate back to Table 1..

## Table 2. Long description

The table compares vital signs and lab values between early IV-to-oral transition (n = 133) and late IV-to-oral transition (n = 92) groups at baseline and 72 hours after antimicrobial therapy initiation. The table has 14 rows and 5 columns. The columns are labeled as Early IV-to-Oral Transition, Late IV-to-Oral Transition, and P. The rows are grouped into Baseline and 72 h after antimicrobial therapy initiation. Baseline measurements include Temperature >38°C or <36°C, HR greater than 90 BPM, RR greater than 20 BPM, WBC in cells/μL, and Lactate in mg/dL. 72 h after antimicrobial therapy initiation measurements include the same parameters. Row 1: Temperature >38°C or <36°C, n, (%), Early IV-to-Oral Transition: 69 (51.9), Late IV-to-Oral Transition: 42 (45.7), P: .358. Row 2: HR greater than 90 BPM, n, (%), Early IV-to-Oral Transition: 81 (60.9), Late IV-to-Oral Transition: 62 (67.4), P: .320. Row 3: RR greater than 20 BPM, n, (%), Early IV-to-Oral Transition: 64 (48.1), Late IV-to-Oral Transition: 58 (63), P: .027. Row 4: WBC in cells/μL, median [IQR], Early IV-to-Oral Transition: 12.4 [9.3–17.4], Late IV-to-Oral Transition: 14.8 [10.8–20.5], P: .011. Row 5: Lactate in mg/dL, median [IQR], Early IV-to-Oral Transition: 1.9 [1.3–3.1]*, Late IV-to-Oral Transition: 2.4 [1.7–4.1]*, P: .001. Row 6: Temperature >38°C or <36°C, n, (%), Early IV-to-Oral Transition: 4 (3), Late IV-to-Oral Transition: 2 (2.2), P: 1.000. Row 7: HR greater than 90 BPM, n, (%), Early IV-to-Oral Transition: 24 (18), Late IV-to-Oral Transition: 23 (25), P: .207. Row 8: RR greater than 20 BPM, n, (%), Early IV-to-Oral Transition: 18 (13.5), Late IV-to-Oral Transition: 31 (33.7), P: <.001. Row 9: WBC in cells/μL, median [IQR], Early IV-to-Oral Transition: 8.9 [6.3–10.5], Late IV-to-Oral Transition: 9.6 [7.6–13.4], P: .002. Row 10: Lactate in mg/dL, median [IQR], Early IV-to-Oral Transition: No recorded values for any patients at 72 h, Late IV-to-Oral Transition: No recorded values for any patients at 72 h.

Navigate back to Table 2..

## Table 3. Long description

A table comparing antibiotic prescriptions between early and late IV-to-oral transition groups. The table has 13 rows and 5 columns. The columns are labeled ‘Early IV-to-oral transition (n = 133)’, ‘Late IV-to-oral transition (n = 92)’, and ‘P’. The rows are labeled with different antibiotics and their prescription counts. Row 1: Intravenous antibiotics prescribed, n (%). Row 2: Multiple antibiotics, 55 (41.4), 63 (68.5), .007. Row 3: Ceftriaxone, 69 (51.9), 28 (30.4). Row 4: Piperacillin-tazobactam, 4 (3), 1 (1.1). Row 5: Aztreonam, 2 (1.5), 0 (0). Row 6: Ertapenem, 1 (0.8), 0 (0). Row 7: Ciprofloxacin, 1 (0.8), 0 (0). Row 8: Ampicillin-sulbactam, 1 (0.8), 0 (0). Row 9: Oral antibiotics prescribed, n (%). Row 10: Cephalosporin, 35 (26.3), 43 (46.7), .010. Row 11: TMP/SMX, 44 (33.1), 17 (18.5). Row 12: Fluoroquinolone, 32 (24.1), 17 (18.5). Row 13: Penicillin, 15 (11.3), 13 (14.1). Row 14: Multiple antibiotics, 7 (5.3), 2 (2.2).

Navigate back to Table 3..

## Table 4. Long description

A table comparing primary and secondary outcomes between early and late IV-to-oral transition groups. The table has 11 rows and 4 columns. Column headers are Early IV-to-oral transition (n = 133), Late IV-to-oral transition (n = 92), and P. Row labels include Primary outcome and Secondary outcomes. Primary outcome rows are Treatment failure within 30 d, n (%), Escalation of/additional antibiotic therapy, Infection-related readmission, Microbiologic relapse, and All-cause mortality. Secondary outcome rows are Hospital length of stay, median [IQR], Duration of treatment, median [IQR], Oral antibiotic duration, Intravenous antibiotic duration, and Total antibiotic duration. Values are provided for each row under the respective columns. Notable trends include shorter median hospital length of stay and total antibiotic duration in the early transition group.

Navigate back to Table 4..
