# Supplementary material for: Early versus late transition to oral antibiotics in Enterobacterales bacteremia
Source: Antimicrob Steward Healthc Epidemiol. 2026 Sep 29;6(1):e300. doi: 10.1017/ash.2026.10886 (PMC13760429; doi:10.1017/ash.2026.10886)
Supplement: Supplementary file 1 [file S2732494X26108869sup001.docx]

| **Potential Risk Factors for**  **Treatment Failure** | **Unadjusted OR**  **(95% CI)** | **P** |
| --- | --- | --- |
| Early Switch to Oral Antibiotics | 0.92 (0.31-2.74) | 1 |
| Age 70 or Greater | 1.01 (0.33-3.13) | 0.982 |
| Male | 0.57 (0.17-1.88) | 0.411 |
| White | 3.43 (0.44-26.9) | 0.312 |
| Diabetes | 0.4 (0.11-1.46) | 0.172 |
| Renal Disease | 0.81 (0.17-3.78) | 1 |
| COPD | 0.52 (0.11-2.41) | 0.528 |
| BMI 35 or Greater | 0.83 (0.22-3.1) | 1 |
| CCI 3 or Greater | 1.15 (0.25-5.38) | 1 |
| ID Consult | 1.08 (0.35-3.33) | 1 |
| ICU Admission | 0.35 (0.08-1.61) | 0.235 |
| Baseline WBC 12 or Greater | 0.86 (0.29-2.58) | 0.785 |

Table S1: Bivariate Analysis
